# Chameleonic Nature of A*β*: Implications for Alzheimer's and Other Amyloid Diseases

**DOI:** 10.1002/bies.70039

**Published:** 2025-07-11

**Authors:** Birgit Strodel

**Affiliations:** ^1^ Institute of Biological Information Processing Structural Biochemistry (IBI‐7) Forschungszentrum Jülich Jülich Germany; ^2^ Institute of Theoretical and Computational Chemistry Heinrich Heine University Düsseldorf Düsseldorf Germany

**Keywords:** Alzheimer's disease, amyloid aggregation, conformational energy landscape, polymorphism, energy landscape–amyloid disease relationship

## Abstract

The amyloid‐*β* peptide (A*β*), implicated in Alzheimer's disease, exhibits significant polymorphism. At the monomer level, A*β* can adopt disordered, helical, and *β*‐hairpin structures, influenced by environmental conditions. Both oligomeric and fibrillar states, characterized by the prevalence of *β*‐sheets, are polymorphic in the arrangement of *β*‐strands. This chameleon‐like behavior arises from A*β*’s unique sequence and relatively flat energy landscape, which facilitates aggregation and may contribute to the prevalence of Alzheimer's disease, while also enabling disaggregation, thus slowing disease progression. In contrast, Creutzfeldt‐Jakob disease, which is much rarer, progresses far more rapidly, likely due to the steeper energy landscape of the prion protein.

## Graphical abstract

The energy landscape of Aβ reveals its flexible, “chameleon‐like” behavior, enabling diverse structures from disordered to β‐rich forms. This polymorphism facilitates aggregation, contributing to Alzheimer's disease progression, while the flat landscape also allows disaggregation, potentially slowing disease progression compared to prion proteins with steeper landscapes.

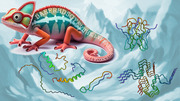

## 1 **A** *β* Sequence: Contrasting Amino Acid Compositions Between N‐ and C‐Terminal Regions

Amyloid‐*β* is a peptide that plays a crucial role in the development of Alzheimer's disease (AD), primarily through its aggregation into toxic assemblies ranging from oligomers to fibrillar plaques in the brain [1]. A*β* peptides can vary in length, with the most common forms being 40 and 42 amino acids long, with the 42‐amino‐acid variant often being more prone to aggregation and associated with AD. In the following, I will refer to these peptides as A*β*40 and A*β*42, respectively, while A*β* will be used to denote both A*β*40 and A*β*42 collectively.

**FIGURE 1:**
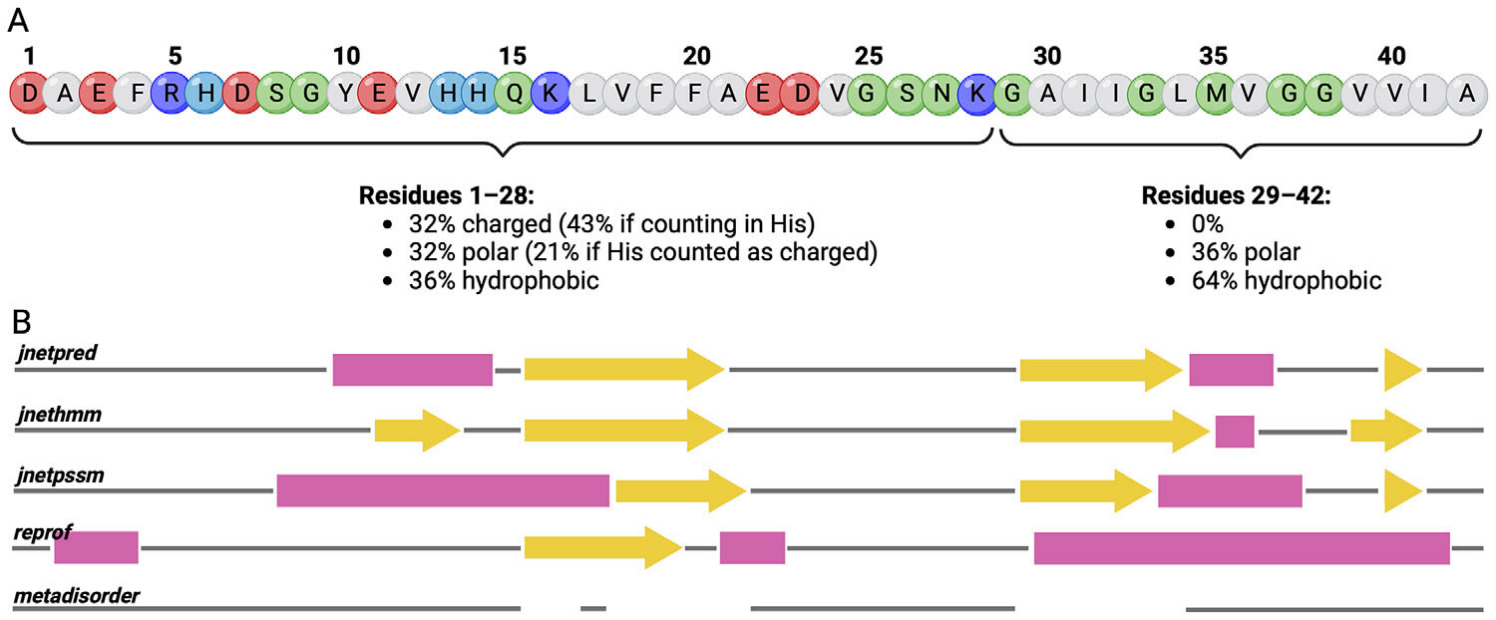
Sequence and secondary structure prediction for A*β*42. (A) The residues are colored according to their physicochemical properties at physiological pH: hydrophobic, gray; polar, green; positively charged, blue (with His in light blue, as it can also be neutral at pH∼7); negatively charged, red. The amino acid composition for residues 1–28 and 29–42 is provided below the sequence. (B) Secondary structure predictions obtained from various prediction methods [4, 5, 6], with *α*‐helices, *β*‐strands, and disordered regions being indicated by magenta squares, yellow arrows, and gray lines, respectively.

The sequence of A*β*42 (Figure 1A) reveals significant differences between the N‐ and C‐terminal regions of the peptide. The N‐terminal side (residues 1 to 28) contains many charged and polar residues, while the C‐terminal side (residues 29 to 42) is predominantly composed of hydrophobic residues. According to Uversky and colleagues, proteins can be categorized as folded or unfolded based on their mean hydrophobicity and net charge [2]. For a neutral protein, if the mean hydrophobicity falls below ∼40%, the protein is usually unfolded. For residues 1–28 of A*β*, the net charge ranges from −3 to 0—depending on the charge states of the histidine residues at positions 6, 13, and 14—and the mean hydrophobicity is 36%. Thus, the N‐terminal region aligns with the characteristics of intrinsically disordered proteins (IDPs). This conclusion is further supported by analyzing the relative enrichment and depletion of specific amino acids. In IDPs, amino acids such as Glu, Ser, Gln, and Lys are significantly enriched, whereas Ala and Gly show moderate enrichment compared to folded proteins; Asp, Thr, and Arg are equally distributed [3]. Approximately 64% of the first 28 A*β* residues fall into these eight amino acid types, suggesting that a majority of the N‐terminal sequence is IDP‐like. In contrast, the C‐terminal region (residues 29 to 42) lacks any charged residues and has 64% hydrophobic content among its 14 residues, reflecting an amino acid composition typical of folded proteins. Most notably, it contains only six residues that are not enriched in ordered proteins, namely four Gly and two Ala residues. Based on these characteristics, one could anticipate a bipartite peptide structure, with the N‐terminal side being disordered and the C‐terminal side adopting a folded conformation.

A more detailed analysis of the sequence, however, indicates that this conclusion does not fully hold, as some hydrophobic residues in the N‐terminal half are clustered in a specific region between residues 17 and 21. Various secondary structure predictions [4, 5] (Figure 1B) show that this peptide region has a high propensity to form a *β*‐strand. Additionally, three of the prediction methods (JnetPred, JnetPSSM, and reprof) suggest the formation of helices of varying lengths N‐terminal to the L17–A21 region. For the C‐terminal portion, four out of five prediction methods indicate that this segment is primarily folded, although the predicted amounts of helical and *β*‐strand structures vary. The predictions generally agree that the first ten N‐ terminal residues and the region E22–K28 are predominantly disordered. The disorder prediction method MetaDisorder [6] forecasts that almost the entire peptide is disordered, with exceptions for the hydrophobic regions L17–A21 and G29–L34. This sequence analysis suggests that reliable structure predictions for the A*β* sequence are challenging, making it difficult to determine whether it is (partly) disordered or folded, and if folded, whether it adopts a helical or *β*‐sheet structure.

## 2 A*β* Monomer in Aqueous Solution: A Disordered Peptide

Over the past 25 years, molecular dynamics (MD) simulations of A*β* have confirmed the challenging nature of secondary structure predictions for this peptide [7]. Depending on the force field used, A*β* structures may exhibit helices, *β*‐sheets, or primarily disordered forms [8]. For example, the Amber99SB‐based force fields [9, 10] a99SB‐UCB [11, 12], a99SB‐ILDN/TIP4P‐D [13], and a99SB‐disp [14] produce expanded and disordered A*β*40 conformations, with a bias toward PPII conformations in the case of a99SB‐disp [8]. In contrast, a99SB‐ILDN/TIP3P [15] results in a more compact A*β*40 due to excessive *β*‐sheet formation, while the Amber force field a03ws [16] tends to trap A*β*40 in highly compact, helical states despite increased peptide‐water interactions. The latest CHARMM force field, Charmm36 (or Charmm36m) [17], generates largely extended, disordered conformations but with a bias toward *β*‐hairpin structures. In fact, the modeling of disordered A*β* structures became feasible only after force fields, originally developed for folded proteins, were modified for IDPs. Using IDP‐adapted force fields and experimental methods such as nuclear magnetic resonance (NMR) spectroscopy, small‐angle x‐ray scattering (SAXS), and fluorescence resonance energy transfer (FRET), it is now established that A*β* in aqueous solution at pH ∼7 is a mostly disordered and rather expanded peptide characterized as a random coil (RC) with an average end‐to‐end distance of 4.3 nm and fast relaxation times ranging from approximately 3 ns for localized backbone motions to 100 ns for global chain relaxation [8, 18, 19, 20].

**FIGURE 2:**
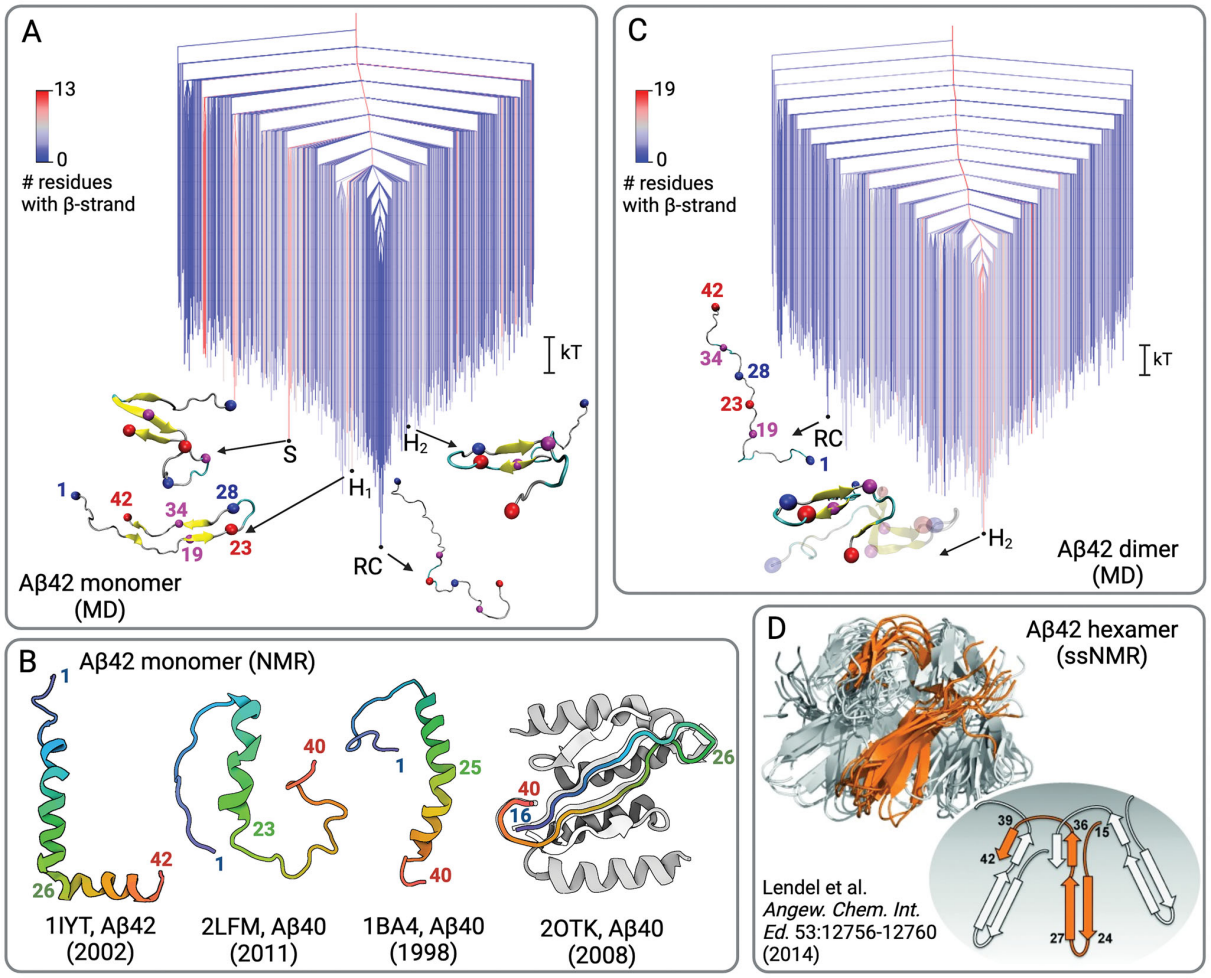
Structures and free energy surfaces of A*β* monomers and oligomers. (A) The FES of the A*β*42 monomer in aqueous solution, illustrated as a disconnectivity graph obtained from MD, reveals a funnel leading to disordered RC states at the bottom. Partially folded conformations are represented as excited states, including *β*‐hairpin states (H_1_ and H_2_) and an S‐shaped state. The energy along the vertical axis is shown in units of kT, and the coloring of the branches corresponds to the number of residues in *β*‐strand conformation for the respective conformation, ranging from 0 (blue) to 13 (red), as indicated by the scale on the left. Key residues are highlighted by spheres: N‐terminues at D1 (blue), F19 (magenta), D23 (red), K28 (blue), L34 (magenta), C‐terminus at A42 (red), as indicated in structure H_1_. (B) Changes in the environment can cause A*β* to adopt folded structures, which, depending on the external conditions, can be either helical or a *β*‐hairpin conformation. The corresponding PDB ID is provided below each conformation, and relevant residues are indicated. (C) The FES of the A*β*42 dimer in aqueous solution, illustrated as a disconnectivity graph obtained from MD, reveals a folding funnel leading to the *β*‐hairpin state H_2_ (which is the same conformation H_2_ as in Panel A). The RC state is considered an excited state within the FES of the dimer. For further explanations regarding the FES representation, see Panel (A); the number of residues in a *β*‐hairpin conformation per peptide in the dimer can reach 19. (D) Model of an A*β*42_CC_ hexamer determined by ssNMR. On the left, the superposition of the ten best models (with residues 1–14 excluded) is shown. On the right, a simplified representation of the hexamer topology is provided, along with the numbering of key residue positions. The *β*‐hairpin corresponds to the conformation obtained from MD for the monomer and dimer (see Panels A and C) and for A*β*40 interacting with an affibody (see Panel B), resulting in an antiparallel *β*‐sheet signature in the oligomers. Panels A and C were adapted with permission from ref. [21] and Panel D from ref. [22].

The free energy surfaces (FESs) derived from MD simulations, some of them guided by NMR data, display a primary funnel leading to the global minimum corresponding to the RC state (Figure 2A) [21, 23, 24]. Excited state conformations, such as a *β*‐hairpin typical of A*β* oligomers or S‐shaped conformations that are the building blocks of fibrils, can be transiently accessed from the RC configurations on the microsecond time scale [21, 24]. This arrangement of the FES, with (partially) folded states at the top of the funnel and disordered states at the bottom, has been referred to as an “inverted free energy landscape” or a “funnel to disorder” [21, 23, 24]. A recent NMR study confirms that A*β* harbors lowly populated transient *β*‐sheet structures, identifying an antiparallel intramolecular *β*‐sheet for the C‐terminal residues I32–A42 linked by a turn at G37 and G38 [25]. Meanwhile, MD simulations suggest that an alternative *β*‐hairpin linking the N‐terminal and C‐terminal hydrophobic regions L17–A21 and G29–L34, stabilized by the salt bridge D23–K28, is also possible [21].

Ultimately, both NMR and MD agree that the monomeric A*β* is predominantly disordered, aligning with the secondary structure predictions made by MetaDisorder (Figure 1B). It is important to note that A*β* does not exhibit glass‐like behavior [21, 24], a characteristic that was previously speculated to be present in IDPs [26]. Such behavior would imply a multifunnel FES and switching conformations over extended time scales [26]. It was suggested that the relatively short sequence of A*β* may be insufficient for glass formation, or that the single glutamine residue at position 15 does not provide the necessary stickiness to induce such behavior [24]. However, considering that in the meantime the aromatic tripeptide YYY has been demonstrated to form an amorphous glass involving water molecules [27], the current conclusion is that peptide length is not the limiting factor. Instead, it is rather the sequence that prevents A*β* from forming a glass.

## 3 A*β* Monomer: Shifts in Environment Induce Diverse Helical and *Β*‐Hairpin Structures

The excited states of A*β* in aqueous solution at pH ∼7, which feature *α*‐helical or *β*‐hairpin structures, may become the most stable conformations upon environmental changes. An examination of the Protein Data Bank (PDB) reveals a predominance of helical A*β* structures determined by solution NMR spectroscopy. Notable examples include the structures with IDs 1IYT for A*β*42 [28], 2LFM for A*β*40 [29], and 1BA4 for A*β*40 [30], all of which are categorized as primarily or partially helical (Figure 2B), consistent with predictions made by various secondary structure prediction methods (Figure 1B). The structure 1IYT was derived from a mixture of 1,1,1,3,3,3‐Hexafluoro‐2‐propanol (HFIP) and water in an 80% HFIP:20% H_2_O v/v ratio. The hydrogen bonds (H‐bonds) between proteins and HFIP are weaker than those formed with water, which likely promotes intraprotein H‐bonds and facilitated the development of the helical structure in A*β*42. Structure 2LFM was obtained for A*β*40 dissolved in a buffer containing 20 mM potassium phosphate and 50 mM NaCl at pH 7.3, utilizing a 93% H_2_O/7% D_2_O solution. Chemical shifts and nuclear Overhauser effects (NOEs) indicated that residues H13 to D23 likely adopt a 3_10_ helical structure, with the terminal residues F4 and G38 packing against the central residues V18/A21 and F19, respectively. This structure formation could be facilitated by the presence of D_2_O which strengthens the intrapeptide H‐bonds while weakening those between peptide and D_2_O. The structure 1BA4 was obtained for A*β*40 in a sodium dodecyl sulfate (SDS) solution (90% H_2_O) at pH 5.1, revealing a helical conformation for the majority of the molecule (residues 15–36), with a kink at residues 25–27 that may function as a hinge between the two helical segments. A similar helix‐kink‐helix structure was identified in another NMR study of A*β*40, conducted in a 100 mM SDS solution with 20 mM sodium phosphate buffer at pH 7–7.6, suggesting that this conformation is robust within SDS micelles [31].

MD simulations revealed a comparable helical structure for A*β*42 when interacting with a lipid cluster [32]. However, these simulations also yielded *β*‐sheet structures, contingent on the peptide's interactions with the lipids. Interestingly, no monomeric A*β* structures featuring *β*‐sheets are available in the PDB. However, a recent time‐resolved solid‐state NMR (ssNMR) study, using a combination of rapid mixing to initiate a structural evolution process, rapid freezing to trap intermediate states, and low‐ temperature ssNMR technology with sensitivity enhancements from dynamic nuclear polarization (DNP), identified for A*β*40 a highly populated *β*‐strand conformation at pH 7.4 before oligomerization occurs [33]. This conformation is U‐shaped or hairpin‐like, bringing the F19 sidechain in proximity with sidechains of L34 and/or M35 (like in conformation H_1_ in Figure 2A). In another MD simulation study, this *β*‐hairpin conformation was induced by the presence of a glycosaminoglycan (GAG) molecule, despite a lack of direct intermolecular interactions [34]. There, the *β*‐hairpin formation appeared to result from descreening of intrapeptide electrostatic interactions, as sodium ions moved away from the peptide toward the negatively charged GAG. A similar *β*‐hairpin structure was elucidated through solution NMR for A*β*40 while in complex with a phage‐display selected affibody protein, where the hairpin, composed of residues 17–36, is stabilized by enclosing both predominantly nonpolar faces within a large hydrophobic tunnel‐like cavity formed by the affibody (PDB ID 2OTK, Figure 2B) [35].

This examination of monomeric A*β* structures could be further expanded, given the extensive literature on the topic. However, it is sufficient to conclude that subtle environmental changes, such as transitioning from pure H_2_O to solvent mixtures containing D_2_O, HFIP, SDS, or other molecules as well as changes in salt concentration, temperature, or peptide concentration influence the sensitive balance between intrapeptide and peptide–environment interactions and can guide the A*β* peptide toward regions of its energy landscape where (partially) folded structures are favored over the RC state.

## 4 A*β* Oligomers: Anti‐Parallel Beta‐Sheets Upon Self‐Assembly

The disorder‐to‐order transition observed in A*β* under varying solution conditions can also be initiated by the peptide's self‐assembly into oligomers. For instance, over a decade ago, a study combining rapid fluorescence techniques with slower two‐dimensional ssNMR revealed a *β*‐hairpin structure formed by the strands E11–D23 and K28–V36, with the most notable interaction occurring between F19 and L34, which serves as a key structural motif of A*β*40 oligomers [36]. Recent NMR studies have confirmed such *β*‐hairpin or U‐shaped structures in A*β*40 oligomers. For example, Barnes et al. conducted solution NMR experiments in which oligomerization of A*β*40 was triggered by a rapid drop in pressure from 2.5 kbar to 1 bar, resulting in oligomer formation in less than 1 s at a concentration of 1.3 mM [37]. This process was accompanied by ordering in residues 16–22 and 29–36. The earlier‐mentioned time‐resolved ssNMR measurements, which identified a *β*‐hairpin structure for the A*β*40 monomer, highlighted prominent intrapeptide contacts between F19 and L34 (or M35) and interpeptide contacts between V18 and G33, which both form on the millisecond time scale during oligomerization [33]. Additionally, infrared (IR) spectroscopy concluded that these *β*‐hairpins assemble into antiparallel *β*‐sheets, showing that A*β*42 oligomers become more homogeneous when the aggregation time increases [38].

Using MD simulations, my lab recently demonstrated how the free energy funnel leading to disorder in the A*β*42 monomer transitions into a folding funnel (Figure 2C), facilitated by the binding of A*β*42 to the hydrophobic region of another A*β*42 peptide [21]. The initial conformational change, which transforms the relatively extended A*β*42 conformation into a hairpin‐like structure, is primarily driven by the formation of a salt bridge between D23 and K28, followed by the establishment of hydrophobic contacts between the strands on either side of the turn, specifically residues L17VFFA21 and A30IIGLMV36. The emergence of these intrapeptide contacts occurs cooperatively with the formation of interpeptide interactions between the hydrophobic regions of both peptides. Once the positions of these hydrophobic contacts are optimized, hydrogen bonds form between the strands, completing the formation of the *β*‐hairpin. A structure for this *β*‐hairpin as part of an A*β* hexamer was provided by a ssNMR study of A*β*42_CC_ where alanine residues at positions 21 and 30 were replaced by cysteines, so that a disulfide bond locks the peptide in a conformation that is incompatible with fibril formation and aggregation is therefore arrested at the oligomeric state (Figure 2D)[22, 39].

Based on these findings, there seems to be a consensus that for small oligomers (*n*‐mers with *n* < 10), the *β*‐hairpin structure is a characteristic element of A*β* oligomers. This could lead to the conclusion that the oligomer state of A*β* is less polymorphic than both the monomer and fibrillar states. However, two comments are in place here. First, the assembly of this hairpin can be manifold and also quite disordered [39]. Second, the transient nature of the oligomers makes it very difficult to capture them and elucidate their structure. Hence, it is very likely that other A*β* oligomer structures exist beyond those discussed here, particularly for the larger *n*‐mers.

## 5 **A** *β* Fibrils: A Zoo of Structures and Interactions

**FIGURE 3:**
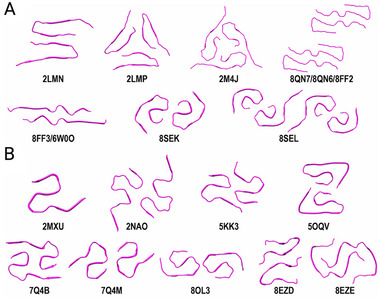
A*β* fibril structures formed by (A) A*β*40 and (B) A*β*42 (with PDB codes given below the structures). Adapted with permission from ref. [40].

An examination of the PDB reveals a wide variety of fibril structures determined for A*β*40 and A*β*42, predominantly characterized by ssNMR or cryogenic electron microscopy (cryo‐EM). In a recent review, Baek and Lee categorized and described these different fibrils, creating a comprehensive fibril atlas, with their summary figure reproduced here as Figure 3 [40]. The common characteristic of these fibrils is that each protofibril exclusively features parallel *β*‐sheets. Beyond this commonality, the fibrils exhibit differences in their physical appearance, such as variations in width and helical twists, which arise from differing backbone conformations, sidechain orientations, and interactions among protofibrils. The specific type of fibril structure formed depends on several growth conditions, including the pH and temperature, the application of agitation, whether the fibrils were grown in vitro or extracted from brain samples, and for those grown in vitro, whether they were seeded from existing fibrils and what types of fibrils were used as seeds. Additionally, the presence of any cofactors that may influence fibril growth, either in vitro or within brain tissue, also plays a role.

Regarding the residues involved in *β*‐sheet formation and intra‐ as well as intermolecular contacts, it is not surprising to see the previously mentioned hydrophobic regions L17–A21 and A30–V36, along with the key salt bridge D23–K28, frequently implicated. However, unlike A*β* oligomers, in which these residues appear to be the sole key structural components, some fibrils contain *β*‐sheets in additional regions, including the N‐terminal region D1–Y10, which was previously thought to be entirely disordered. For example, in the A*β*40 fibril structure with PDB ID 8QN7, the structure is stabilized by electrostatic and polar interactions involving residues H6, S8, E11, H13, and K16 [41]. A salt bridge formed between E11 and K16 stabilizes a turn in the peptide, similar to the D23–K28 interaction often seen further down the sequence. A comprehensive list of all interactions in various fibril structures can be found in the work of Baek and Lee [40].

In comparing the structures of monomers and oligomers of A*β*40 and A*β*42, few differences were observed between the peptides. However, for fibrils, the two additional residues at the C‐terminus of A*β*42 are significant. Not only do they increase the hydrophobicity of the peptide, but they also elongate the peptide, allowing for alternative fibril conformations. These extra residues facilitate a turn in the C‐terminal region between M35 and V39—which can also be found in the monomer (see conformation H_2_ in Figure 2A and ref. [25])—enabling the formation of S‐shaped fibrils not observed in A*β*40 [40]. This S‐shaped conformation is further stabilized by various hydrophobic interactions involving the C‐terminal residues, as well as electrostatic interactions arising from the negative charge at the C‐terminus of A*β*42. Regardless of whether they are formed by A*β*40, A*β*42, or their mutants, all fibrils share the common feature that two or more protofibrils can pack against each other, involving diverse peptide interfaces.

Further information about the relevance of the different residues for the A*β* fibril formation was obtained from a cell‐based assay, which enabled the massively parallel quantification of how sequence variations affect the peptide aggregation [42]. It revealed that mutations within the hydrophobic C‐terminal region (29–42), particularly residues 33–38, significantly disrupt aggregation, identifying this region as likely central to the nucleation transition state. Conversely, many substitutions in the more IDP‐like N‐terminal region (1–28) accelerated aggregation, especially after introducing polar (N, H, T, Q) or positively charged (K, R) residues. This suggests that reducing A*β*’s net charge promotes aggregation, which is negatively charged at physiological pH. The E22 position is notably significant due to familial mutations such as the Arctic (E22G), Osaka (E22∆), Dutch (E22Q), and Italian (E22K) mutations, which are all linked to early‐onset familial Alzheimer's disease (FAD) and show accelerated aggregation in the assay. Some FAD mutations exhibit distinct structural differences from wild‐type A*β* fibrils connected to sporadic Alzheimer's disease [40]. Uniquely, the Iowa mutation (D23N) results in antiparallel amyloid fibrils (PDB 2LNQ) [43]. While data on mutant oligomer structures are limited, FAD‐linked mutations like E22G are known to markedly enhance oligomer formation [44].

Similar to mutations, pH significantly affects A*β* aggregation by altering electrostatic interactions. A study showed no A*β*42 fibrillization at pH 3.5 and 4.5, whereas the strongest amyloid signals were observed at pH 7.4 and 8.0, with moderate fibrillization at pH 5.6 and 6.5, and slower assembly at pH 5.4 and 9.5 [45]. Notably, amyloid fibril and oligomer formation can be inversely related; a study reported an 8000‐fold increase in A*β*42 oligomerization as pH decreased from 7.4 (extracellular) to 4.8 (endo‐lysosomal), correlating with reduced fibril formation [46]. This is biologically relevant because A*β* accumulates at low pH in neuronal endo‐lysosomal vesicles. Lipids and other co‐factors further influence A*β* aggregation and fibril structures. For example, A*β* can form phospholipid‐containing biomolecular condensates on bilayers, accelerating amyloid nucleation [47]. Cryo‐EM and NMR findings indicate that lipids bind to fibrils when grown with lipids, resulting in structures similar to brain‐ seeded fibrils, highlighting the biological importance of A*β*–lipid interactions [48]. This is further supported by a recent study on the in‐tissue structure of A*β* in fresh post‐mortem AD donor brain [49]. Using various cryo‐EM and cryo‐tomography techniques, a mixture of fibrils and protofilaments in parallel arrays and lattice‐like structures was identified, incorporating non‐amyloid components such as extracellular vesicles, droplets, and open lipid bilayer sheets [49].

The structural diversity observed in A*β* fibrils can only occur if the resulting fibrils possess similar thermodynamic charac‐ teristics. Indeed, free energy calculations of amylin fibrils revealed that all structures considered exhibit similar per‐residue energy scores, making them equally likely from the thermodynamic viewpoint [50]. While thermodynamic stability dictates which structures are feasible, the relative population of each fibril structure is influenced not by its stability but by the rate at which it is formed [50, 51]. This was demonstrated for amylin using cryo‐EM at various time points during in vitro fibrillization [50]. The study found that fibrils formed during the lag, growth, and plateau phases exhibited different structures of similar thermodynamic stability, with new forms appearing and others disappearing as fibrillization progressed. Nonetheless, the final fibril structures observed in this study are somewhat more thermodynamically stable than the earlier ones [50], making them more resistant against fragmentation in the presence of shear forces. Whether the same principles apply to A*β* fibril formation remains to be investigated.

## 6 Conclusions: Polymorphism and the Energy Landscape of A*β* and Implications for Amyloid Diseases

A*β* is demonstrated to be polymorphic at the levels of the monomer, oligomer, and fibril. Starting with the inherent vagueness of secondary structure predictions, A*β*’s sequence confers the potential to adopt various conformations: it can be disordered— particularly in the N‐terminal half with an IDP‐like amino acid composition—adopt a helical structure in regions 17–21 and 30–34, where hydrophobic residues cluster, or form a *β*‐hairpin structure enabled by residues 23–28 that facilitate a turn between the hydrophobic stretches, allowing them to adopt strand‐like geometries rather than helical conformations. These different structures are nearly equally probable, and depending on environmental conditions, one conformation may become more favorable than another. This structural indecisiveness, combined with the clustering of hydrophobic residues and potential for various salt bridges, not only allows A*β* to undergo amyloid aggregation but also to adopt numerous fibrillar structures. In summary, akin to a chameleon that changes color based on its environment, A*β* dynamically alters its structure.

The polymorphic nature of A*β* is closely linked to its neurotoxicity and AD development. For small oligomers, toxicity increases with size: dimers are about three times, while trimers and tetramers are 8‐ and 13‐fold more toxic than monomers, correlating with higher *β*‐sheet content [52]. Notably, antiparallel *β*‐sheet oligomer, such as those stabilized through the above mentioned A*β*42_CC_, [22, 39] are exceptionally toxic, being 50 times more neurotoxic than fibrils or wild‐type A*β*42, which only forms transient oligomers [53]. A recent study analyzing soluble A*β* oligomers from eight brain regions revealed diverse sizes and structures, with smaller oligomers (∼2 nm diameter, less than 100 nm in length) from hippocampal extracts showing the highest potency [54]. Their toxicity involves membrane disruption, calcium dysregulation, receptor blockade, and impaired neurotransmission, ultimately leading to synaptic dysfunction [55]. It is important to note that the comparison between synthetic and brain‐derived A*β* oligomers remains an active area of research; differences in receptor binding suggest notable in vivo–in vitro variations [56, 57]. Concerning fibril polymorphism and AD progression, fibrils associated with sporadic Alzheimer's disease tend to exhibit distinct folds and morphologies compared to those linked to early‐onset familial Alzheimer's disease. Additionally, the corresponding amyloid plaque deposits differ in diffusivity, focality, and localization, further influencing their pathogenic roles [58].

Another consequence of the chameleon‐like behavior of A*β* is that this peptide poses a particularly challenging target for therapeutic approaches due to the absence of a definitive structure associated with AD. This complexity helps explain the slow progress in developing drugs for AD [59]. To date, three anti‐A*β* antibodies—Aduhelm (aducanumab), Kisunla (donanemab), and Leqembi (lecanemab)—have been approved by the FDA. While aducanumab and donanemab primarily target fibrils [60], lecanemab has been shown to reduce A*β* protofibrils and is the first to demonstrate slowing of cognitive decline in early‐stage AD [61]. However, these therapies are costly and associated with significant toxic side effects. Consequently, there is ongoing research to develop small molecules capable of clearing A*β* aggregates. Two promising candidates are: ALZ‐801 (homotaurine prodrug, phase 3) [62], which prevents A*β* oligomer formation [63]; and PRI‐002 (all D‐ptlhthnrrrrr peptide, phase 2) [64], which inhibits oligomerization via electrostatic interactions between its five arginines and the E22/D23 region of A*β*, blocking *β*‐hairpin formation necessary for toxic oligomerization [65].

A hypothesis arising from the structural flexibility of A*β* suggests that the relative ease with which this peptide assumes the (pre)fibrillar state may account for the prevalence of AD once A*β* reaches a critical concentration in the brain, which typically occurs with increasing age. This can be attributed to its relatively flat energy landscape. However, this not only facilitates aggregation but also the dissociation of A*β* aggregates. Moreover, as aggregation appears to be a frequent event, the body can develop mechanisms to counteract such aggregation, such as chaperones [66]. If this were not the case, AD, which progresses over years or even decades, might develop much more rapidly.

**FIGURE 4:**
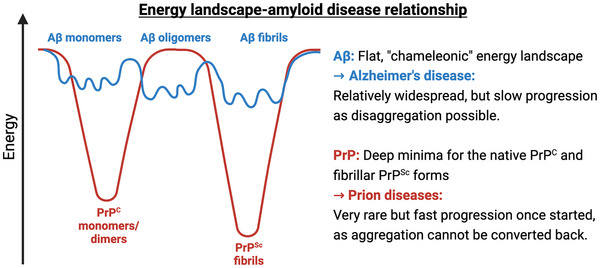
Hypothesis on the relationship between the energy landscape and corresponding amyloid disease. The energy landscapes shown should be interpreted qualitatively rather than quantitatively. Additionally, the exclusion of PrP oligomers in this figure should not be construed as them being irrelevant; this omission is primarily for simplification purposes.

In contrast, consider another amyloid disease, such as Creutzfeldt‐Jakob disease (CJD), which, while rare, leads to rapid mortality [67]. The average survival time following a CJD diagnosis is typically only a few months. The prion protein primarily exists in a folded state with *α*‐helices, accompanied by an unstructured N‐terminal region [68]. Although this flexible, unstructured segment allows for some mobility, the remainder of the prion protein remains stably folded, requiring significant energy to misfold into the amyloid state, where the majority of *α*‐helices are transformed into *β*‐sheets [69]. However, once this free energy barrier is overcome, the progression toward the amyloid state appears to follow a steep downhill, nonreversible trajectory in the free energy landscape, as otherwise the fast disease progression could not be explained. Thus, the flatness of the A*β* energy landscape underlying its chameleon‐like behavior presents not only disadvantages but also advantages. This leads me to propose the relationship between the energy landscape and amyloid disease as summarized in Figure 4.

## Conflicts of Interest

The authors declare no potential conflicts of interest.

## Data Availability

Data sharing is not applicable to this article as no datasets were generated or analyzed during the current study.

## References

[1] F. Chiti and C. M. Dobson , “Protein Misfolding, Amyloid Formation, and Human Disease: A Summary of Progress Over the Last Decade,” Annual Review of Biochemistry 86, no. 1 (2017): 27–68, 10.1146/annurev-biochem-061516-045115. 28498720

[2] A. K. Dunker , C. J. Oldfield , J. Meng , et al., “The Unfoldomics Decade: An Update on Intrinsically Disordered Proteins,” BMC Genomics 9, no. Suppl 2 (2008), 10.1186/1471-2164-9-s2-s1. PMC2559873 18831774

[3] V. N. Uversky , “The Alphabet of Intrinsic Disorder,” Intrinsically Disordered Proteins 1, no. 1 (2013): 24684, 10.4161/idp.24684. PMC5424795 28516010

[4] A. Drozdetskiy , C. Cole , J. Procter , and G. J. Barton , “JPred4: A Protein Secondary Structure Prediction Server,” Nucleic Acids Research 43, no. W1 (2015): W389–W394, 10.1093/nar/gkv332. 25883141 PMC4489285

[5] B. Rost and C. Sander , “Prediction of Protein Secondary Structure at Better Than 70% Accuracy,” Journal of Molecular Biology 232, no. 2 (1993): 584–599, 10.1006/jmbi.1993.1413. 8345525

[6] L. P. Kozlowski and J. M. Bujnicki , “MetaDisorder: A Meta‐Server for the Prediction of Intrinsic Disorder in Proteins,” BMC Bioinformatics 13, no. 1 (2012): 111, 10.1186/1471-2105-13-111. 22624656 PMC3465245

[7] H. Fatafta , M. Khaled , B. Kav , O. O. Olubiyi , and B. Strodel , “A Brief History of Amyloid Aggregation Simulations,” WIREs: Computational Molecular Science 14, no. 1 (2024): 1703, 10.1002/wcms.1703.

[8] A. Paul , S. Samantray , M. Anteghini , M. Khaled , and B. Strodel , “Thermodynamics and Kinetics of the Amyloid‐*β* Peptide Revealed by Markov state Models Based on MD Data in Agreement With Experiment,” Chemical Science 12, no. 19 (2021): 6652–6669, 10.1039/d0sc04657d. 34040740 PMC8132945

[9] R. B. Best and G. Hummer , “Optimized Molecular Dynamics Force Fields Applied to the Helix−Coil Transition of Polypeptides,” Journal of Physical Chemistry B 113, no. 26 (2009): 9004–9015, 10.1021/jp901540t. 19514729 PMC3115786

[10] K. Lindorff‐Larsen , S. Piana , K. Palmo , et al., “Improved Side‐Chain Torsion Potentials for the Amber ff99SB Protein Force Field,” Proteins 78, no. 8 (2010): 1950–1958, 10.1002/prot.22711. 20408171 PMC2970904

[11] P. S. Nerenberg and T. Head‐Gordon , “Optimizing Protein−Solvent Force Fields to Reproduce Intrinsic Conformational Preferences of Model Peptides,” Journal of Chemical Theory and Computation 7 (2011): 1220–1230, 10.1021/ct2000183. 26606367

[12] P. S. Nerenberg , B. Jo , C. So , A. Tripathy , and T. Head‐Gordon , “Optimizing Solute–Water van der Waals Interactions To Reproduce Solvation Free Energies,” Journal of Physical Chemistry B 116 (2012): 4524–4534, 10.1021/jp2118373. 22443635

[13] S. Piana , A. G. Donchev , P. Robustelli , and D. E. Shaw , “Water Dispersion Interactions Strongly Influence Simulated Structural Properties of Disordered Protein States,” Journal of Physical Chemistry B 119 (2015): 5113–5123. 25764013 10.1021/jp508971m

[14] P. Robustelli , S. Piana , and D. Shaw , “Developing a Molecular Dynamics Force Field for Both Folded and Disordered Protein States,” Proceedings National Academy of Science USA 115, no. 21 (2018): E4758–E4766, 10.1073/pnas.1800690115. PMC6003505 29735687

[15] W. L. Jorgensen , J. Chandrasekhar , J. D. Madura , R. W. Impey , and M. L. Klein , “Comparison of Simple Potential Functions for Simulating Liquid Water,” Journal of Chemical Physics 79, no. 2 (1983): 926–935, 10.1063/1.445869.

[16] R. B. Best , W. Zheng , and J. Mittal , “Balanced Protein–Water Interactions Improve Properties of Disordered Proteins and Non‐Specific Protein Association,” Journal of Chemical Theory and Computation 10, no. 11 (2014): 5113–5124, 10.1021/ct500569b. 25400522 PMC4230380

[17] J. Huang , S. Rauscher , G. Nawrocki , et al., “CHARMM36m: An Improved Force Field for Folded and Intrinsically Disordered Proteins,” Nature Methods 14 (2017): 71–73, 10.1038/nmeth.4067. 27819658 PMC5199616

[18] J. Roche , Y. Shen , J. H. Lee , J. Ying , and A. Bax , “Monomeric Aβ 1–40 and Aβ 1–42 Peptides in Solution Adopt Very Similar Ramachandran Map Distributions That Closely Resemble Random Coil,” Biochemistry 55, no. 5 (2016): 762–775, 10.1021/acs.biochem.5b01259. 26780756 PMC4750080

[19] F. Meng , M. M. Bellaiche , J. Y. Kim , G. H. Zerze , R. B. Best , and H. S. Chung , “Highly Disordered Amyloid‐*β* Monomer Probed by Single‐Molecule FRET and MD Simulation,” Biophysical Journal 114, no. 4 (2018): 870–884, 10.1016/j.bpj.2017.12.025. 29490247 PMC5984999

[20] S. Acharya , K. Srivastava , S. Nagarajan , and L. Lapidus , “Monomer Dynamics of Alzheimer Peptides and Kinetic Control of Early Aggregation in Alzheimer's Disease,” Chemphyschem: A European Journal of Chemical Physics and Physical Chemistry 17 (2016): 3470–3479, 10.1002/cphc.201600706. 27490673 PMC5806154

[21] M. Schäffler , D. J. Wales , and B. Strodel , “The Energy Landscape of A*β*42: A Funnel to Disorder for the Monomer Becomes a Folding Funnel for Self‐Assembly,” Chemical Communications 60, no. 92 (2024): 13574–13577, 10.1039/d4cc02856b. 39479923

[22] C. Lendel , M. Bjerring , A. Dubnovitsky , et al., “A Hexameric Peptide Barrel as Building Block of Amyloid‐β Protofibrils,” Angewandte Chemie International Edition 53, no. 47 (2014): 12756–12760, 10.1002/anie.201406357. 25256598

[23] D. Granata , F. Baftizadeh , J. Habchi , et al., “The Inverted Free Energy Landscape of an Intrinsically Disordered Peptide by Simulations and Experiments,” Scientific Reports 5, no. 1 (2015): 15499, 10.1038/srep15449. 26498066 PMC4620491

[24] D. Chakraborty , J. E. Straub , and D. Thirumalai , “Energy Landscapes of A*β* Monomers Are Sculpted in Accordance With Ostwald's Rule of Stages,” Science Advances 9, no. 12 (2023): eadd6921, 10.1126/sciadv.add6921. 36947617 PMC10032606

[25] T. Kakeshpour , V. Ramanujam , C. A. Barnes , Y. Shen , J. Ying , and A. Bax , “A Lowly Populated, Transient *β*‐Sheet Structure in Monomeric A*β*1‐42 Identified by Multinuclear NMR of Chemical Denaturation,” Biophysical Chemistry 270 (2021): 106531, 10.1016/j.bpc.2020.106531. 33453683 PMC7878406

[26] B. Strodel , “Energy Landscapes of Protein Aggregation and Conformation Switching in Intrinsically Disordered Proteins,” Journal of Molecular Biology 433, no. 20 (2021): 167182, 10.1016/j.jmb.2021.167182. 34358545

[27] G. Finkelstein‐Zuta , Z. A. Arnon , T. Vijayakanth , et al., “A Self‐Healing Multispectral Transparent Adhesive Peptide Glass,” Nature 630, no. 8016 (2024): 368–374, 10.1038/s41586-024-07408-x. 38867128

[28] O. Crescenzi , S. Tomaselli , R. Guerrini , et al., “Solution Structure of the Alzheimer Amyloid β‐Peptide (1–42) in an Apolar Microenvironment,” European Journal of Biochemistry 269, no. 22 (2002): 5642–5648, 10.1046/j.1432-1033.2002.03271.x. 12423364

[29] S. Vivekanandan , J. R. Brender , S. Y. Lee , and A. Ramamoorthy , “A Partially Folded Structure of Amyloid‐Beta(1–40) in an Aqueous Environment,” Biochemical and Biophysical Research Communications 411, no. 2 (2011): 312–316, 10.1016/j.bbrc.2011.06.133. 21726530 PMC3148408

[30] M. Coles , W. Bicknell , A. A. Watson , D. P. Fairlie , and D. J. Craik , “Solution Structure of Amyloid β‐Peptide(1−40) in a Water−Micelle Environment. Is the Membrane‐Spanning Domain Where We Think It Is?,” Biochemistry 37, no. 31 (1998): 11064–11077, 10.1021/bi972979f. 9693002

[31] J. Jarvet , J. Danielsson , P. Damberg , M. Oleszczuk , and A. Gräslund , “Positioning of the Alzheimer Aβ(1–40) Peptide in SDS Micelles Using NMR and Paramagnetic Probes,” Journal of Biomolecular NMR 39, no. 1 (2007): 63–72, 10.1007/s10858-007-9176-4. 17657567

[32] H. Fatafta , B. Kav , B. F. Bundschuh , J. Loschwitz , and B. Strodel , “Disorder‐to‐Order Transition of the Amyloid‐*β* Peptide Upon Lipid Binding,” Biophysical Chemistry 280 (2022): 106700, 10.1016/j.bpc.2021.106700. 34784548

[33] J. Jeon , W. M. Yau , and R. Tycko , “Early Events in Amyloid‐*β* Self‐Assembly Probed by Time‐Resolved Solid State NMR and Light Scattering,” Nature Communications 14, no. 1 (2023): 2964, 10.1038/s41467-023-38494-6. PMC10205749 37221174

[34] M. Schäffler , S. Samantray , and B. Strodel , “Transition Networks Unveil Disorder‐to‐Order Transformations in A*β* Caused by Glycosaminoglycans or Lipids,” International Journal of Molecular Sciences 24, no. 14 (2023): 11238, 10.3390/ijms241411238. 37510997 PMC10380057

[35] W. Hoyer , C. Grönwall , A. Jonsson , S. Ståhl , and T. Härd , “Stabilization of a β‐Hairpin in Monomeric Alzheimer's Amyloid‐β Peptide Inhibits Amyloid Formation,” Proceedings National Academy of Science USA 105, no. 13 (2008): 5099–5104, 10.1073/pnas.0711731105. PMC2278213 18375754

[36] B. Sarkar , V. S. Mithu , B. Chandra , et al., “Significant Structural Differences Between Transient Amyloid‐β Oligomers and Less‐Toxic Fibrils in Regions Known To Harbor Familial Alzheimer′s Mutations,” Angewandte Chemie International Edition 53, no. 27 (2014): 6888–6892, 10.1002/anie.201402636. 24756858

[37] C. A. Barnes , A. J. Robertson , J. M. Louis , P. Anfinrud , and A. Bax , “Observation of *β*‐Amyloid Peptide Oligomerization by Pressure‐Jump NMR Spectroscopy,” Journal of the American Chemical Society 141, no. 35 (2019): 13762–13766, 10.1021/jacs.9b06970. 31432672 PMC9357264

[38] F. Vosough and A. Barth , “Characterization of Homogeneous and Heterogeneous Amyloid‐*β*42 Oligomer Preparations With Biochemical Methods and Infrared Spectroscopy Reveals a Correlation Between Infrared Spectrum and Oligomer Size,” ACS Chemical Neuroscience 12, no. 3 (2021): 473–488, 10.1021/acschemneuro.0c00642. 33455165 PMC8023574

[39] M. Khaled , I. Rönnbäck , L. L. Ilag , A. Gräslund , B. Strodel , and N. Österlund , “A Hairpin Motif in the Amyloid‐*β* Peptide Is Important for Formation of Disease‐Related Oligomers,” Journal of the American Chemical Society 145, no. 33 (2023): 18340–18354, 10.1021/jacs.3c03980. 37555670 PMC10450692

[40] Y. Baek and M. Lee , “Exploring the Complexity of Amyloid‐Beta Fibrils: Structural Polymorphisms and Molecular Interactions,” Biochemical Society Transactions 52, no. 4 (2024): 1631–1646, 10.1042/bst20230854. 39034652

[41] Y. Yang , A. G. Murzin , S. Peak‐Chew , et al., “Cryo‐EM Structures of A*β*40 Filaments From the Leptomeninges of Individuals With Alzheimer's Disease and Cerebral Amyloid Angiopathy,” Acta Neuropathologica Communications 11, no. 1 (2023): 191, 10.1186/s40478-023-01694-8. 38049918 PMC10694933

[42] M. Seuma , B. Lehner , and B. Bolognesi , “An Atlas of Amyloid Aggregation: The Impact of Substitutions, Insertions, Deletions and Truncations on Amyloid Beta Fibril Nucleation,” Nature Communication 13, no. 1 (2022): 7084, 10.1038/s41467-022-34742-3. PMC9674652 36400770

[43] W. Qiang , W. M. Yau , Y. Luo , M. P. Mattson , and R. Tycko , “Antiparallel *β*‐Sheet Architecture in Iowa‐Mutant *β*‐Amyloid Fibrils,” Proceedings National Academy of Science USA 109, no. 12 (2012): 4443–4448, 10.1073/pnas.1111305109. PMC3311365 22403062

[44] M. Lu , N. Williamson , A. Mishra , et al., “Structural Progression of Amyloid‐*β* Arctic Mutant Aggregation in Cells Revealed by Multiparametric Imaging,” Journal of Biological Chemistry 294, no. 5 (2019): 1478–1487, 10.1074/jbc.ra118.004511. 30504224 PMC6364760

[45] S. Kobayashi , Y. Tanaka , M. Kiyono , et al., “Dependence pH and Proposed Mechanism for Aggregation of Alzheimer's Disease‐Related Amyloid‐β(1–42) Protein,” Journal of Molecular Structure 1094 (2015): 109–117, 10.1016/j.molstruc.2015.03.023.

[46] M. P. Schützmann , F. Hasecke , S. Bachmann , et al., “Endo‐Lysosomal A*β* Concentration and pH Trigger Formation of A*β* Oligomers That Potently Induce Tau Missorting,” Nature Communication 12, no. 1 (2021): 4634, 10.1038/s41467-021-24900-4. PMC8324842 34330900

[47] G. Šneideriene˙ and S. D. Adhikari , “Lipid‐Induced Condensate Formation From the Alzheimer's A*β* Peptide Triggers Amyloid Aggregation,” Proceedings National Academy of Science USA 122, no. 4 (2025): e2401307122, 10.1073/pnas.2401307122. PMC11789053 39854227

[48] B. Frieg , M. Han , K. Giller , et al., “Cryo‐EM Structures of Lipidic Fibrils of Amyloid‐*β* (1‐40),” Nature Communications 15, no. 1 (2024): 1297, 10.1038/s41467-023-43822-x. PMC10864299 38351005

[49] M. A. G. Gilbert , N. Fatima , J. Jenkins , et al., “CryoET of *β*‐Amyloid and Tau Within Postmortem Alzheimer's Disease Brain,” Nature 631, no. 8022 (2024): 913–919, 10.1038/s41586-024-07680-x. 38987603 PMC11269202

[50] M. Wilkinson , Y. Xu , D. Thacker , et al., “Structural Evolution of Fibril Polymorphs During Amyloid Assembly,” Cell 186, no. 26 (2023): 5798–5811, 10.1016/j.cell.2023.11.025. 38134875 PMC7617692

[51] W. Qiang , K. Kelley , and R. Tycko , “Polymorph‐Specific Kinetics and Thermodynamics of *β*‐Amyloid Fibril Growth,” Journal of the American Chemical Society 135, no. 18 (2013): 6860, 10.1021/ja311963f. 23627695 PMC3686096

[52] K. Ono , M. M. Condron , and D. B. Teplow , “Structure–Neurotoxicity relationships of amyloid β‐Protein oligomers,” Proceedings of the National Academy of Sciences 106, no. 35 (2009): 14745–14750, 10.1073/pnas.0905127106. PMC2736424 19706468

[53] A. Sandberg , L. M. Luheshi , S. Söllvander , et al., “Stabilization of Neurotoxic Alzheimer Amyloid‐*β* Oligomers by Protein Engineering,” Proceedings of the National Academy of Sciences 107, no. 35 (2010): 15595–15600, 10.1073/pnas.1001740107. PMC2932621 20713699

[54] D. I. Sideris , J. S. H. Danial , D. Emin , et al., “Soluble Amyloid Beta‐Containing Aggregates Are Present throughout the Brain at Early Stages of Alzheimer's Disease,” Brain Communications 3, no. 3 (2021): fcab147, 10.1093/braincomms/fcab147. 34396107 PMC8361392

[55] A. B. Reiss , H. A. Arain , M. M. Stecker , N. M. Siegart , and L. J. Kasselman , “Amyloid Toxicity in Alzheimer's Disease,” Reviews in the Neurosciences 29, no. 6 (2018): 613–627, 10.1515/revneuro-2017-0063. 29447116

[56] L. M. Smith , M. A. Kostylev , S. Lee , and S. M. Strittmatter , “Systematic and Standardized Comparison of Reported Amyloid‐β Receptors Sufficiency, Affinity, and Alzheimer's Disease Relevance,” Journal of Biological Chemistry 294, no. 15 (2019): 6042–6053, 10.1074/jbc.ra118.006252. 30787106 PMC6463724

[57] K. Al Adem and S. Lee , “Structural Polymorphism and Cytotoxicity of Brain‐Derived β‐Amyloid Extracts,” Protein Science 32, no. 5 (2023): 4639, 10.1002/pro.4639. PMC10127262 37051675

[58] Y. Yang , D. Arseni , W. Zhang , et al., “Cryo‐EM Structures of Amyloid‐*β* 42 Filaments From Human Brains,” Science 375, no. 6577 (2022): 167–172, 10.1126/science.abm7285. 35025654 PMC7612234

[59] A. L. Boxer and R. Sperling , “Accelerating Alzheimer's Therapeutic Development: The Past and Future of Clinical Trials,” Cell 186, no. 22 (2023): 4757–4772, 10.1016/j.cell.2023.09.023. 37848035 PMC10625460

[60] J. Cummings , A. M. L. Osse , D. Cammann , and J. Powell , and J. Chen , “Anti‐Amyloid Monoclonal Antibodies for the Treatment of Alzheimer's Disease,” Biodrugs 38, no. 1 (2023): 5–22, 10.1007/s40259-023-00633-2. 37955845 PMC10789674

[61] C. H. van Dyck , C. J. Swanson , and P. Aisen , “Lecanemab in Early Alzheimer's Disease,” New England Journal of Medicine 388, no. 1 (2023): 9–21, 10.1056/nejmoa2212948. 36449413

[62] S. Abushakra , A. P. Porsteinsson , M. Sabbagh , et al., “APOLLOE4 Phase 3 Study of Oral ALZ‐801/Valiltramiprosate in APOE *ε*4/*ε*4 Homozygotes With Early Alzheimer's Disease: Trial Design and Baseline Characteristics,” Translational Research & Clinical Interventions 10, no. 3 (2024): e12498, 10.1002/trc2.12498. PMC11322500 39144121

[63] P. Kocis , M. Tolar , J. Yu , et al., “Elucidating the A*β*42 Anti‐Aggregation Mechanism of Action of Tramiprosate in Alzheimer's Disease: Integrating Molecular Analytical Methods, Pharmacokinetic and Clinical Data,” CNS Drugs 31, no. 6 (2017): 495–509, 10.1007/s40263-017-0434-z. 28435985 PMC5488121

[64] J. Kutzsche , N. C. Cosma , G. Kauselmann , et al., “Oral PRI‐002 Treatment in Patients With MCI or Mild AD: A Randomized, Double‐Blind Phase 1b Trial,” Nature Commun 16, no. 1 (2025), 10.1038/s41467-025-59295-z. PMC12053642 40324978

[65] O. Olubiyi , D. Frenzel , D. Bartnik , et al., “Amyloid Aggregation Inhibitory Mechanism of Arginine‐Rich D‐Peptides,” Current Medicinal Chemistry 21, no. 12 (2014): 1448–1457, 10.2174/0929867321666131129122247. 24304283

[66] A. Wentink , C. Nussbaum‐Krammer , and B. Bukau , “Modulation of Amyloid States by Molecular Chaperones,” Cold Spring Harbor Perspectives in Biology 11, no. 7 (2019): a033969, 10.1101/cshperspect.a033969. 30755450 PMC6601462

[67] K. K. Sitammagari and W. Masood , Creutzfeldt Jakob Disease (StatPearls Publishing, 2025). 29939637

[68] R. Zahn , A. Liu , T. Lührs , et al., “NMR Solution Structure of the human Prion Protein,” Proceedings National Academy of Science USA 97, no. 1 (2000): 145–150, 10.1073/pnas.97.1.145. PMC26630 10618385

[69] M. Sanz‐Hernández , J. D. Barritt , J. Sobek , S. Hornemann , A. Aguzzi , and A. De Simone , “Mechanism of Misfolding of the human Prion Protein Revealed by a Pathological Mutation,” Proceedings National Academy of Science USA 118, no. 12 (2021): e2019631118, 10.1073/pnas.2019631118. PMC7999870 33731477

